# Rehabilitation of an irradiated marginal mandibulectomy patient using immediately loaded basal implant-supported fixed prostheses and hyperbaric oxygen therapy: A 2-year follow-up

**DOI:** 10.1016/j.ijscr.2020.05.018

**Published:** 2020-05-21

**Authors:** Fadia Awadalkreem, Nadia Khalifa, Abdelnasir G. Ahmad, Ahmed Mohamed Suliman, Motaz Osman

**Affiliations:** a Assistant Professor, Department of Oral Rehabilitation, Prosthodontics Division, Faculty of Dentistry, University of Khartoum, Sudan; b Assistant Professor, Chair of the Department of Preventive and Restorative Dentistry, University of Sharjah/Faculty of Dental Medicine, Sharjah, Sharjah, United Arab Emirates; c Associate Professor, International University of Africa, Oral and Maxillofacial Surgery Department, Khartoum, Sudan; d Professor, Department of Oral and Maxillofacial Surgery, Faculty of Dentistry, University of Khartoum, Khartoum, Sudan; e Consultant, Implant Department, Khartoum Teaching Dental Hospital, Federal Ministry of Heath, Khartoum, Sudan

**Keywords:** Mandibular resection, Basal implant-supported prostheses, Hyperbaric Oxygen, Immediately loaded fixed prostheses

## Abstract

**Introduction:**

The prosthetic rehabilitation of mandibular defects owing to tumor resection is challenging, especially when the patient has undergone subsequent radiotherapy.

**Presentation of case:**

A 46-year old male presented with a marginal mandibular resection. Following surgery, the patient received adjunctive radiation therapy with a total dose of 70 grays. On clinical examination, the patient presented with severely resorbed edentulous jaws, with an anterior marginal mandibular resection and an obliterated vestibular sulcus. The panoramic radiograph showed a hypocellularity of the maxillary and mandibular bones. A multidisciplinary team was formed, and a treatment plan was formulated which involved the construction of a vestibuloplast stent, and the application of 20 hyperbaric oxygen sessions before implant treatment and 10 more sessions after implant insertion. A total of 16 basal cortical screw implants were inserted to support the fixed prostheses, and a vestibuloplasty was performed to improve esthetics. No complications were observed, and at the 2-year follow-up, the patient presented with excellent peri-implant soft tissue health; increased bone-implant contact; and stable, well-functioning prostheses.

**Discussion:**

The construction of a stable, retentive, well-supported removable prosthesis may be complicated in cases of comprehensive mandibular resection. Basal implants can eliminate the need for bone grafting, and reduce the treatment period required for providing a fixed prosthesis.

**Conclusion:**

To our knowledge this is the first evidence reporting the use of fixed basal implant-supported prostheses in irradiated bone, in conjunction with hyperbaric oxygen therapy. A treatment modality that significantly improves the peri-implant tissue health, and ensures an excellent implant-bone contact.

## Highlights

• Mandibulectomy after oral cancer resection can cause severe facial disfigurement.
• The use of adjunctive radiotherapy may compromise the success rate of reconstructive implant therapy.
• Hyperbaric oxygen therapy (HBO) can repair tissue damage after radiotherapy.
• Basal implants reduces risk of peri-implantitis and osteoradionecrosis.
• This report describes HBO and basal implant treatment for marginal mandibulectomy.
• HBO and basal implants are successful treatment modalities for these patients.

## 1 Introduction

Mandibulectomy following oral cancer resection can result in a severe facial disfigurement with multiple disabilities, such as impaired mastication, phonation, swallowing, and poor salivary and tongue control; these adversely affect patient quality of life [1], [2], [3], [4]. Although surgical eradication is the most commonly used treatment modality for oral cancer, patients with extensive primary and/or late stage cancer may require adjunctive radiotherapy or chemotherapy [3], [5]. The majority of patients with head and neck cancer receive between 50 and 70 grays (Gy) as a therapeutic dose [6].

Radiotherapy affects the tumor cell DNA and alters the molecular properties of tumor cell mutation. Initially, it affects the remodeling activity of the bone cells. At low levels of radiation (<50 Gy), rapidly dividing cells are susceptible to necrosis, while at high doses (>70 Gy), osteocytes cells are devitalized, and fibrosis takes place within the connective tissue [4], [5]. Later, vascular alterations occur in the following cascade: hyperemia, endarteritis, thrombosis, occlusion, and obliteration of the small vessels [5], [6]. Subsequently, the bone marrow undergoes fibrosis and fatty degeneration, which may induce osteoradionecrosis [6]. Previous studies have reported the ability of hyperbaric oxygen therapy (HBO) to effectively repair tissue damage associated with radiotherapy [1], [2], [4], [6], [7], [8], [9], [10], [11].

The use of HBO to prevent osteonecrosis was first introduced by Marx et al. [11] in 1985, who recommended 20–40 preoperative dives once or twice per week, for 60 min each, and 10 postoperative dives [11]. A well-known adjunctive protocol of HBO for tooth extraction or implant therapy is the provision of 20–30 sessions before (each session lasting 90 min) and after (each session lasting 10 min) the treatment; these sessions are conducted at an absolute pressure of 100% oxygen, and at a compression of 2.4 atmospheres [6]. This protocol ensures better wound healing in the implant site, especially when the bone in this region has received more than 50 Gy of radiation [1], [6].

The prosthetic rehabilitation of patients with mandibular resection is extremely challenging due to the severe loss of both hard and soft tissues, reduced vestibular sulcus, diminished salivary flow following radiation, and the psychological status of the patient [1], [2], [7]. Edentulous patients who have undergone a marginal mandibulectomy commonly present with a limited remaining bony structure; this may compromise the retention, support, and stability of a removable reconstructive prosthesis [1], [7]. Thus, the use of dental implants can significantly improve the functional outcome of the prosthesis [1], [2], [6], [7]. Nevertheless, many risk factors may influence the use of dental implants in patients who have undergone radiotherapy, such as patient age, sex, implant site, total radiation dose, time between end of radiotherapy and implant osteotomy, and type of radiation therapy [6], [12], [13], [14], [15]. Furthermore, the use of endosseous implants may necessitate a bone grafting procedure, which can be further complicated with the induction of radiotherapy [16], [17], [18]. On the other hand, the use of basal implants in these patients can be successfully attempted to avoid the bone grafting procedure and its associated complications [16], [17], [18].

Basal implants are designed to utilize the strongest cortical bone available in the jaw [16], [17], [18]. The basal cortical screw (BCS®) implant is a special basal implant design characterized by a (1) minimally invasive crestal insertion approach (flapless technique); (2) smooth surface that eradicates the risk of peri-implantitis and thus eliminates the risk of osteoradionecrosis; and (3) thin guidance tip that ensure the centralization of the implant [16], [17], [18]. Moreover, the BCS® implant has a monoblock design, which minimizes the risk of prosthetic failure associated with the implant-abutment interface. Additionally, implants are splinted using a circular metal framework that enhances the biomechanical distribution of masticatory forces; this facilitates the use of an immediately loaded prosthesis [16], [17], [18].

The main cause of implant failure in irradiated bone is the lack of osseointegration and primary stability [7], [8], [12]. As such, the use of basal implants can be very advantageous in irradiated jaws, owing to the high primary stability achieved by deeply anchoring the horizontal plates of the implants inside the cortical bone [16], [17], [18]. Although the successful use of immediately loaded, fixed, basal implant-supported prostheses has been reported [16], [17], [18], its efficacy in patients who have undergone mandibulectomy and radiotherapy remains unclear. This report is the first to describe the use of HBO and basal implant treatment in a patient with marginal mandibulectomy. This case report complies with the SCARE criteria [19].

## 2 Presentation of case

**Fig. 1:**
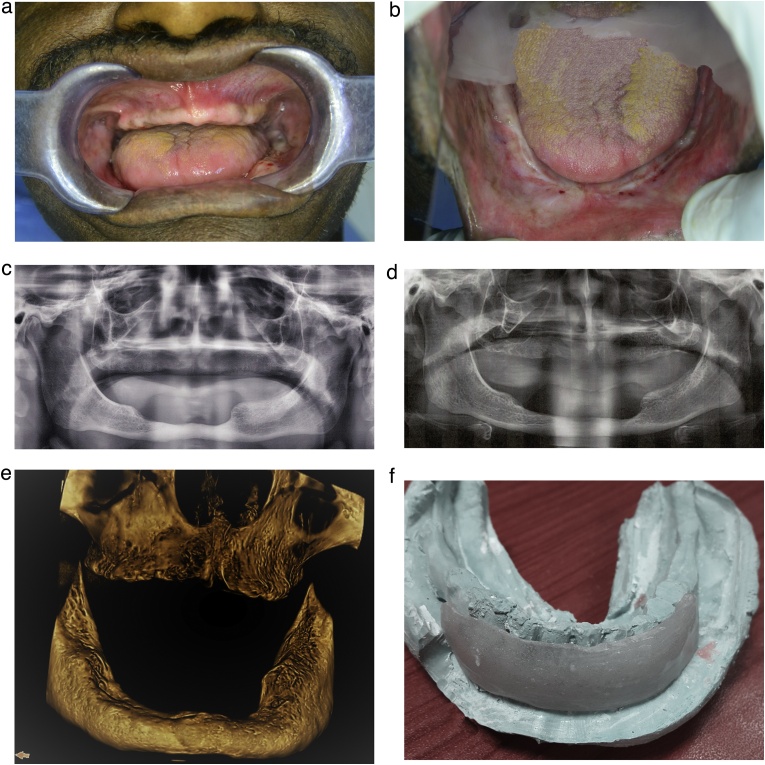
The patient’s clinical presentation. (a) The intraoral view presenting a severely resorbed maxillary edentulous ridge. (b) The intraoral view presenting an anterior marginal resected mandibular bone associated with an obliterated sulcus. (Image was taken using a mirror). (c) The panoramic radiograph shows hypocellularity in the maxilla and mandible. (d) The preoperative panoramic view of the patient after the first 20 sessions of Hyperbaric oxygen. (e) The preoperative 3D view of the maxilla and mandible using cone-beam computed tomography. (f) A photograph showing a heat-cured acrylic stent to be inserted following vestibuloplasty, to ensure the correct repositioning of the muscles.

A 46-year-old male was referred to the Department of Prosthodontics after marginal resection of the mandible, owing to squamous cell carcinoma. The patient underwent radiation sessions (with a total dose of 70 Gy) 16 months following mandibular resection. On clinical examination, the patient presented with severely resorbed edentulous jaws, with an anterior marginal mandibular resection (Fig. 1a, b). The vestibular sulcus of the mandible was obliterated anteriorly (Fig. 1b). A radiographic evaluation of the maxillary and mandibular jaws, using the digital panoramic view (Planmeca ProMax, Finland), showed features of hypocellularity. (Fig. 1c).

A multidisciplinary team was formed to formulate a comprehensive treatment plan, which involved the construction of a vestibuloplasty stent, and the provision of 20 HBO sessions (90 min each) before implant treatment, followed by 10 sessions postoperatively to improve the bony foundation [1,6,7]. This was followed by the insertion of BCS® implants to support the fixed prostheses, and vestibuloplasty to improve the esthetic outcome. The treatment plan was fully discussed with the patient, and informed consent for treatment and publication was obtained. Ethical approval was acquired from the ethical committee of the authors’ institute.

### 2.1 Treatment

Both panoramic and cone-beam computed tomography views were captured following the first 20 sessions of HBO (Fig. 1d, e). A mandibular diagnostic impression was obtained using irreversible hydrocolloid to obtain a diagnostic cast, on which a heat-cured acrylic resin vestibuloplasty stent was constructed (Fig. 1a–f).

**Fig. 2:**
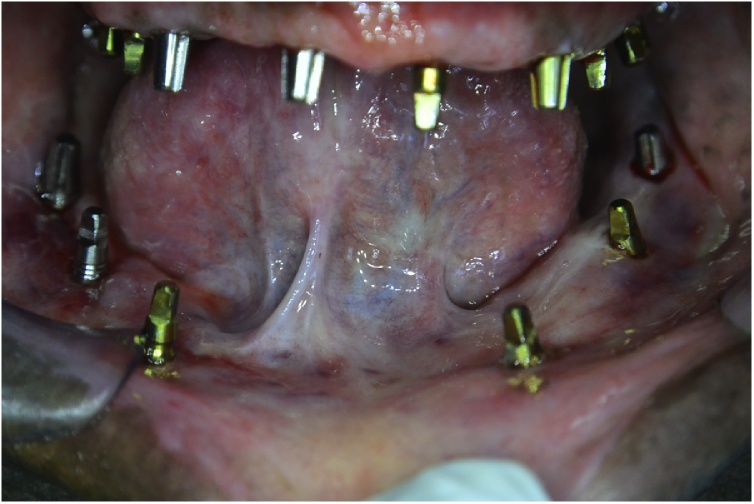
An intraoral clinical photograph presenting the distribution of the 16 BCS® implants: 10 in the maxilla and 6 in the mandible.

The surgical procedures were performed under aseptic conditions and antibiotic prophylaxis. Local anesthesia (lidocaine 2% with adrenaline 1:100000) was injected, and 16 BCS® implants of a suitable length and width were inserted using a flapless technique (Fig. 2).

### 2.2 The definitive fixed basal implant-supported prostheses

**Fig. 3:**
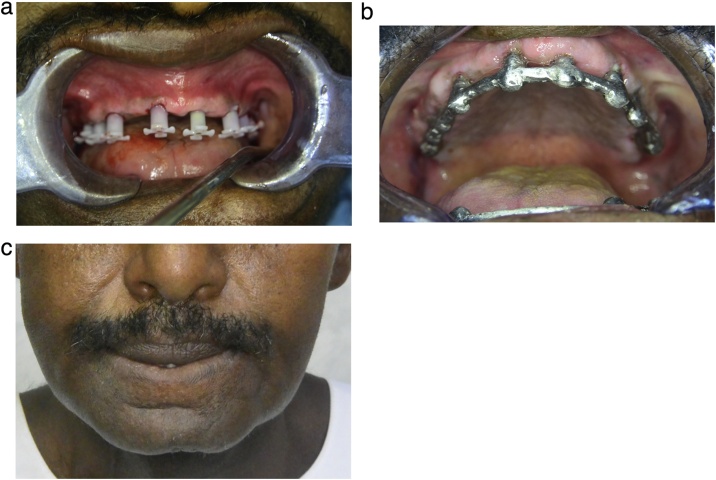
The prosthetic rehabilitation of the patient. (a) The intraoral view illustrating the impression coping secured over the abutments’ head. (b) The metal framework try-in. (c) The frontal view of the patient after the insertion of the final maxillary and mandibular implant-supported prostheses. Lips incompetence and a depression at the mento-labial sulcus region was observed.

**Fig. 4:**
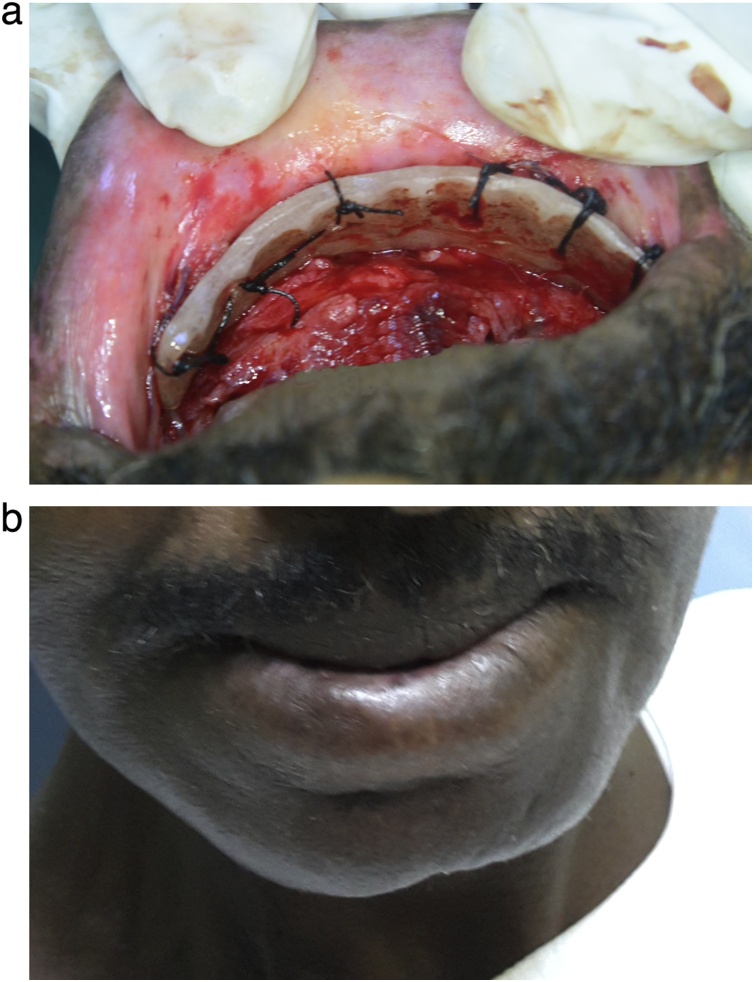
The vestibuloplasty. (a) The intra-oral view depicting the insertion and suturing of the vestibuloplasty stent. (b) The extra-oral view illustrating the insertion of the vestibuloplasty stent.

**Fig. 5:**
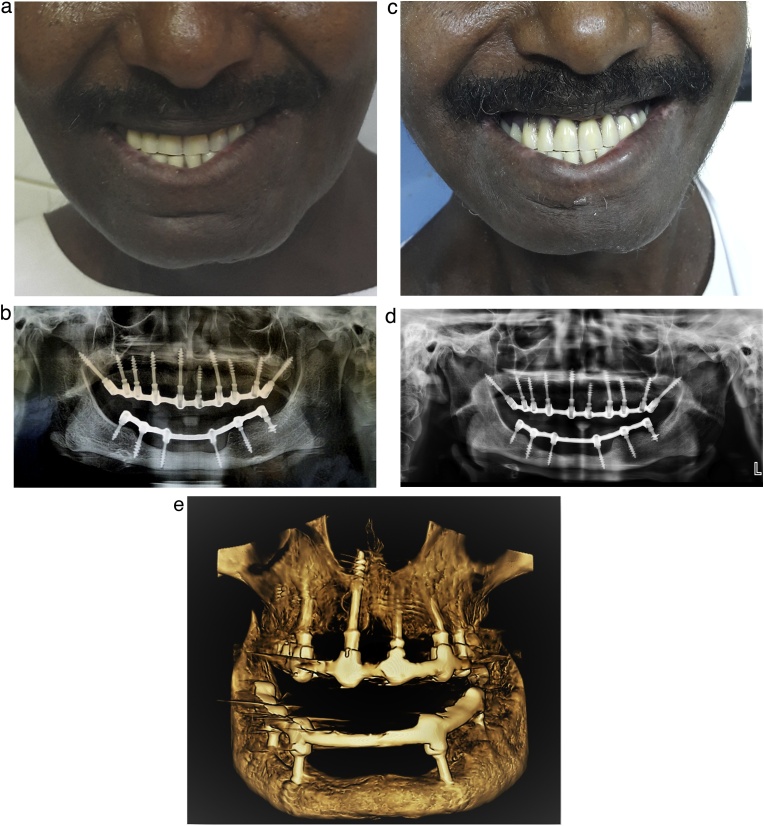
Follow-up images of the immediately loaded basal implant-supported fixed prostheses. (a) The frontal view of the patient after 2 weeks of follow-up, and after the removal of the vestibuloplasty stent. (b) A panoramic radiograph showing the maxillary and mandibular prostheses after 2 weeks of function. (c) The frontal view of the patient at the 2-year follow-up; the patient was highly satisfied with the treatment. (d) A panoramic radiograph showing the maxillary and mandibular prostheses after 2 years of function, showing an excellent peri-implant bone contact. (e) A 3D view of the maxilla and mandible using cone-beam computed tomography after 2 years of function.

Impression copings were secured and the final impression was acquired using silicone impression material (Ivoclar Vivadent AG, Schaan, Liechtenstein) (Fig. 3a). One day later, a metal framework try-in was attempted (Fig. 3b). On the third day, the final acrylic hybrid prostheses were inserted and cemented using Glass Ionomer Luting Cement (Fuji I® GC Corporation, Japan) (Fig. 3c). Postoperative radiographs were obtained (Fig. 3d). A vestibuloplasty was performed at the anterior region of the mandible, and this was accompanied by stent insertion and suturing. (Fig. 4a, b) The patient was provided with oral hygiene instructions and recalled at 2 weeks, and 3, 6, 9, 12, and every 6 months thereafter. Two weeks after complete soft tissue healing, the vestibuloplasty stent was removed, revealing a highly esthetic outcome (Fig. 5a).

Both clinical and radiographic examinations were performed throughout the follow-up visits (Fig. 5b, c, d,e). No complaints were reported by the patient, who noted improvements in his esthetics, chewing efficiency, speech, overall self-esteem, and social life.

## 3 Discussion

Prosthetic rehabilitation of edentulous patients following surgical removal of oral cancer presents with a number of challenging factors [1], [2], [6], [7], [12]. Thus, a multidisciplinary treatment plan is necessary to ensure successful outcomes [1].

Although several prosthetic options have been described for mandibular reconstruction, their ability to maintain and provide an optimal foundation for the retention, stability, and support of a removable prosthesis is unclear [1], [2], [14]. This is especially so in patients presenting with comprehensive post-surgical anatomical alterations and eliminated vestibular sulci. Therefore, in such cases, the use of implants can significantly improve a patient's oral function and self-esteem [1], [2], [7], [13], [14].

Basal implants have several advantages in patients with limited bony support [16], [17], [18]. For example, it eliminates the need for augmentation procedures, and their associated risks. Moreover, these implants reduce the cost of treatment and the time required to provide an immediately functional prosthesis. The smooth surface and small penetrating tip of BCS implants can reduce, or even eliminate the risk of peri-implantitis, thus maximizing implant survival and success rates [16], [17], [18].

According to the literature, the use of a removable, implant-supported overdenture can improve patient oral hygiene [4], [7], [13], [14]. However, in the present case, the use of a hygienic design ensured the same advantages by providing a space for salivary washing actions, and permitting the use of a small interdental brush for cleaning [4], [7]. Moreover, this fixed prosthesis design eliminated the mucosal pain and discomfort that may be associated with mucosal irritation in implant overdentures [4], [7], [13]. Such irritation may result in frictional ulcers and subsequent infection, particularly in irradiated patients [4], [7], [13], [14]. Pjetursson et al. [20] conducted a systematic review of the survival and complication rates of fixed implant-supported prostheses with a mean observation period of 5 years, and reported that such treatment is both safe and predictable.

Many previous studies [1], [2], [4], [6], [8], [9], [10], [11] have reported the ability of HBO to improve implant osseointegration and prevent osteoradionecrosis [1], [2], [4], [6], [8], [9], [10], [11]. In contrast, Schoen et al. [21] reported no advantageous effect for HBO in improving implant survival, compared to the use of prophylactic antibiotics. In the present case, both HBO and antibiotic prophylaxis were used to ensure successful implant treatment and reduce the risk of osteoradionecrosis.

## 4 Conclusion

HBO, in combination with basal implants, is a highly successful treatment modality for patients with head and neck cancer who have a history of radiation therapy.

## Declarations of Competing Interest

None.

## Funding

No funding was obtained for this study.

## Ethical approval

The research was registered at the research centre of the Khartoum Dental Teaching Hospital, Federal Ministry of Health, Khartoum, Sudan, after the approval of the research ethical committee of Khartoum Dental Teaching Hospital.

## Consent

The approval of the patient was obtained for the treatment and publication of this case report.

## Author contributions

Awadalkreem F contributed to the conceptualization, manging the patient, writing, editing, finalization and submission of the case.

Khalifa N contributed to the conceptualization, validation, and supervision of the case.

Ahmad A contributed to the conceptualization, validation, manging the patient, and supervision of the case.

Suliman AM was contributed to the conceptualization validation, and supervision of the case.

Osman M contributed to the conceptualization, manging the patient, editing, and finalization of the manuscript.

## Registration of research studies

The research was registered at the research centre of the Khartoum Dental Teaching Hospital, Federal Ministry of Health, Khartoum, Sudan, after the approval of the research ethical committee of Khartoum Dental Teaching Hospital, Federal Ministry of Health, Khartoum, Sudan.

## Guarantor

Fadia Awadalkreem.

## Provenance and peer review

Not commissioned, externally peer-reviewed.
